# Performance analysis of large language models in the domain of legal argument mining

**DOI:** 10.3389/frai.2023.1278796

**Published:** 2023-11-17

**Authors:** Abdullah Al Zubaer, Michael Granitzer, Jelena Mitrović

**Affiliations:** ^1^Faculty of Computer Science and Mathematics, Chair of Data Science, University of Passau, Passau, Germany; ^2^Group for Human Computer Interaction, Institute for Artificial Intelligence Research and Development of Serbia, Novi Sad, Serbia

**Keywords:** natural language processing (NLP), argument mining, legal data, European Court of Human Rights (ECHR), sequence classification, GPT-4, ChatGPT, large language models

## Abstract

Generative pre-trained transformers (GPT) have recently demonstrated excellent performance in various natural language tasks. The development of ChatGPT and the recently released GPT-4 model has shown competence in solving complex and higher-order reasoning tasks without further training or fine-tuning. However, the applicability and strength of these models in classifying legal texts in the context of argument mining are yet to be realized and have not been tested thoroughly. In this study, we investigate the effectiveness of GPT-like models, specifically GPT-3.5 and GPT-4, for argument mining via prompting. We closely study the model's performance considering diverse prompt formulation and example selection in the prompt via semantic search using state-of-the-art embedding models from OpenAI and sentence transformers. We primarily concentrate on the argument component classification task on the legal corpus from the European Court of Human Rights. To address these models' inherent non-deterministic nature and make our result statistically sound, we conducted 5-fold cross-validation on the test set. Our experiments demonstrate, quite surprisingly, that relatively small domain-specific models outperform GPT 3.5 and GPT-4 in the F1-score for premise and conclusion classes, with 1.9% and 12% improvements, respectively. We hypothesize that the performance drop indirectly reflects the complexity of the structure in the dataset, which we verify through prompt and data analysis. Nevertheless, our results demonstrate a noteworthy variation in the performance of GPT models based on prompt formulation. We observe comparable performance between the two embedding models, with a slight improvement in the local model's ability for prompt selection. This suggests that local models are as semantically rich as the embeddings from the OpenAI model. Our results indicate that the structure of prompts significantly impacts the performance of GPT models and should be considered when designing them.

## 1 Introduction

In recent years, natural language processing (NLP) and artificial intelligence (AI), in general, have seen tremendous popularity in research and application (Chen et al., 2021). Given the success of large language models (LLMs) (Zhao et al., 2023), the progress in NLP is unprecedented. Development of pre-trained language models (PLMs) like BERT (Devlin et al., 2018) and RoBERTa (Liu et al., 2019) has been utilized to achieve state-of-the-art results on diverse benchmark datasets, e.g. GLUE (Wang et al., 2018), SQuAD (Rajpurkar et al., 2016), and RACE (Lai et al., 2017).

The performance of LLMs has surpassed benchmark datasets and demonstrated accomplishments in more day-to-day complex tasks. For example, OpenAI Codex creates Python (Van Rossum and Drake, 2009) functions from docstrings (Chen et al., 2021) and powers GitHub Copilot,^1^ an influential developer companion. LLMs have a substantial impact directly or indirectly on diverse sectors of our life, including but not limited to legal judgment prediction (Chalkidis et al., 2019), education (Zhai, 2022; Kasneci et al., 2023; Lo, 2023), social media (Aljabri et al., 2023), music industry (Ji et al., 2023) and drug discovery (Liu et al., 2021b). These later and aforementioned success reports can be attributed to the development of transformer (Vaswani et al., 2017) networks, now a de-facto neural network architecture adopted for PLMs and LLMs. Moreover, the introduction of Generative pre-trained transformers (GPT) (Radford et al., 2018), especially its successors, GPT-3 (Brown et al., 2020a), GPT-3.5,^2^ ChatGPT.^3^ and GPT-4 (OpenAI, 2023) has significantly impacted both the research and industry communities.

The primary component of these LLMs consists of the decoder block from the transformer architecture (Vaswani et al., 2017) and are trained with the next-token prediction objective (Radford et al., 2018). Whereas PLMs, for instance, BERT (Devlin et al., 2018) consist only of the encoder block from the transformer architecture, with the training objective of predicting masked tokens. One of the critical characteristics of LLMs, for instance, PaLM (Chowdhery et al., 2022), LLaMA-1 (Touvron et al., 2023a), Llama-2 (Touvron et al., 2023b), and Galactica (Taylor et al., 2022) is the ability to perform in-context learning (ICL) (Brown et al., 2020a) based on a few training examples, where we primarily focus on prompting instead of fine-tuning the model. Whereas PLMs follow classical pre-training and fine-tuning techniques as a learning paradigm. ICL is the process through which LLMs can perform a specific task based on human written instruction given as plain text. In essence, given a text sequence (*X*) to the model, the model then produces the next most probable token (*y*) at the current position (*t*) given the previous sequence (*x*_1_, *x*_2_, …, *x*_*t*−1_), where a token can be a word or sub-word. During training, the model is trained to maximize the probability of producing the next token conditioned on the context (Chang et al., 2023).


(1) $$\begin{array}{l} P\left(y|X\right)=P\left(y|x_{1},x_{2},\ldots ,x_{t-1}\right) \end{array}$$

This simple yet effective modeling technique has proven competent in various natural language processing tasks (Brown et al., 2020a). One of the key findings that emerged from the latest advancement of LLMs is a technique described as ICL (Dong et al., 2023; Liu et al., 2023a; Weng, 2023), which can be defined as a conditional text generation problem (Liu et al., 2021a). The central idea behind ICL is to “learn” from given example/s and perform a similar task without any parameter updates through prompts. The complete task is usually provided to the model as a text in natural language with instructions and examples. The fundamental method of ICL still needs to be thoroughly comprehended; it is understood primarily intuitively. This has led to some intriguing studies (Dai et al., 2023; Han et al., 2023; Von Oswald et al., 2023; Zhang et al., 2023b) that attempted to demystify it. Despite this limitation of understanding how exactly ICL functions, there has been a noticeable success with this method (Brown et al., 2020a; Press et al., 2022; Wei et al., 2023; Yao et al., 2023).

Given the success of ICL, several studies have demonstrated that it is not robust against minor changes in the prompt. Zhao et al. (2021a) presented that the performance of ICL is sensitive to the structure of the prompt, the training samples present in the prompt, and the succession in which they are presented. Interestingly, on SST-2 dataset (Socher et al., 2013), just by altering the ordering of the examples in the prompt, the accuracy dropped from 93.4% to 54.3%, while the former was close to the state-of-the-art result (Zhao et al., 2021a). Lu et al. (2022) have reported that the performance of the model degrades as the order of the examples in the prompt changes. In Liu et al. (2021a), the authors conducted an in-depth empirical study of the performance of ICL based on the type of examples selected in the prompt. They have found that the performance of ICL relies strongly on selecting the type of examples based on semantic similarity regarding the test example compared to random selection. They have conducted sentiment analysis on IMDB (Maas et al., 2011), table-to-text generation using ToTTo (Parikh et al., 2020), and question-answering tasks based on Natural Questions (Kwiatkowski et al., 2019), Web Questions (Berant et al., 2013) and Trivia Question Answering (Joshi et al., 2017), and their method of using semantically similar example outperformed random selection. One fundamental observation that we can derive from these investigations is that the steps of constructing an effective prompt or, in other words, Prompt Engineering (White et al., 2023), are the new hyperparameters (Yu and Zhu, 2020) that are needed to be optimized to bring the optimal performance of LLMs.

Likewise, LLMs have made substantial progress in the legal domain (Trautmann et al., 2022; Katz et al., 2023b; Nay et al., 2023; Sun, 2023). Blair-Stanek et al. (2023) investigated if GPT-3 (via the API provided by OpenAI) can perform statutory reasoning on SARA dataset (Holzenberger et al., 2020) and performed better than previous state-of-the-art results that were BERT based. They have studied few-shot prompting (along with its variants) (Brown et al., 2020a) and chain-of-thought (CoT) prompting (Wei et al., 2023). Yu et al. (2022) also investigates CoT on the COLIEE entailment task, which involved the Japanese Civil Code articles, and they found that CoT prompting outperformed the baseline. Choi et al. (2023) analyzed the capability of ChatGPT on law exams and found out that ChatGPT can pass the exam but with low grades.

However, there is no analysis of GPT 3.5 or GPT-4 for legal argument mining, and yet to be adopted for the ECHR dataset. To the best of our knowledge, only one work (Pojoni et al., 2023) explicitly deals with argument mining using the latest GPT model (GPT-4). Likewise, as previously pointed out, GPT-like models are extremely sensitive against prompt structure and need to analyze how their structure impacts these models' performance. Additionally, using local models for prompt selection still needs to be investigated well. Consequently, our work makes the following contributions:

1. We quantify the performance of ChatGPT^4^(GPT-3.5) and GPT-4 (OpenAI, 2023) for argument component classification in the legal domain using, publicly available, ECHR dataset.^5^ Compared to GPT-3.5 and GPT-4, the baseline models had better performance in terms of F1-score for both premise and conclusion classes, with a 1.9% and 12% gain, respectively.
2. We investigate the performance impact of example selection in few-shot prompting. We systematically modify the prompt by using a few-shot learning strategy (Brown et al., 2020b), using 0, 2, 4, and 8-shot examples. For example selection based on semantics similarity and dissimilarity, we utilized two embedding models; an open-source local model, multi-qa-mpnet-base-dot-v1^6^ and text-embedding-ada-002^7^ from the OpenAI. The GPT-3.5 model demonstrates improved performance in the premise recognition task when the prompt comprises semantically similar examples. Our observation demonstrated that there was no significant difference between the two embedding models. Therefore, it points toward the direction of adopting local models in favor of the OpenAI embedding model.
3. We analyzed the prompt and the ECHR dataset, demonstrating that the inherent annotation characteristics of the dataset significantly lowered the performance of GPT-3.5 and GPT-4.
4. We open source the output of GPT-3.5 and GPT-4^8^.

The rest of the paper is structured as follows. Section 2 presents an introduction and related work regarding argument mining in the legal domain and background on the GPT model we have utilized in our experiments. Section 3 presents the materials and methods related to our study. Section 3.1 provides detailed information regarding the ECHR dataset. Section 3.2 discusses the argument mining task we have addressed in our paper. Section 3.3 elaborates on our experimental setup while providing details for reproducing our experiments. Section 3.4 presents the language (Section 3.4.1) and embedding models (Section 3.4.2) we adopted for our experiments. Section 3.4.3 introduces our prompt. Section 4 presents our results and findings, including analysis of prompt structure (Section 4.1) and model's performance (Section 4.2). In Section 4.2.1 and Section 4.2.2, we present a thorough analysis of the prompt and ECHR dataset. Finally, in Section 5, we present our key findings, limitations, and future research directions.

## 2 Related work in argument mining in legal domain and GPT model

Argumentation plays a vital role for professionals working in the legal domain, for example, lawyers (Palau and Moens, 2009). Lawyers must present their thoughts in a structured and argumentative manner to support their views. Therefore, it is evident that identifying the chain of argument that leads to a conclusion will provide a more precise understanding of their decision (Palau and Moens, 2009). Argument mining (AM) is the automated procedure of identifying and extracting the argument elements and the structure from text written in natural language (Lawrence and Reed, 2020). The most adopted model for argument mining in the literature is Walton (2009)'s model for argumentation (Lippi and Torroni, 2016). It models an argument as a statement comprising three parts: a set of premises, one conclusion, and a relation from the premise to the conclusion. Other argumentation models, such as Toulmin's argumentation model (Bentahar et al., 2010), capture complex relationships between argument components. AM comprises several sequential stages (Lippi and Torroni, 2016; Wambsganss et al., 2020). The first stage involves detecting arguments and non-argumentative texts; in the second stage, the argument components are determined; for example, if the latent model is Walton's argumentation model, then the components are claim/premise. In the third stage, the link between the argument components is determined, and, again, based on the underlying model, the kind of relationship is identified. Jointly, all these stages constitute a complete AM pipeline.

AM has been the topic of discussion in the legal domain (which is the focus of our paper) for an extended period of time (Zhang et al., 2022). To the best of our knowledge, the earliest work to automatically recognize argumentative sentences in the legal text, European Court of Human Rights (ECHR)^9^, was carried out by simple classifiers with handcrafted features (Moens et al., 2007). In later work, Mochales and Moens (2008, 2011) have expanded their study on the ECHR dataset, and they have used Support Vector Machine (Noble, 2006) with handcrafted feature in Mochales and Moens (2011).

Notably, most of the recent AM techniques for legal documents involve the AM pipeline mentioned before. For example, Grundler et al. (2022) created a legal corpus annotated for argument mining and performed argument detection and classification. The authors have utilized the classical machine learning method (TF-IDF) for text representation along with transformer-based models (Sentence-BERT and Legal-BERT) coupled with traditional classification models, such as Random Forest and Naive Bayes. In Poudyal et al. (2020); Zhang et al. (2021); Lillis et al. (2022), the authors have approached the AM problem, as we mentioned before, using transformer-based models (elaborated in Section 3.3). This pipeline approach directs to a situation where an error made in the first stage will be carried over to the later tasks. To mitigate this issue, Zhang et al. (2023a) has proposed an alternative view of the AM task, depicting the legal documents as a graph structure. They have approached AM (argument extraction stage) as one unified process using Graph Neural Networks on an improved graph structure by adding virtual nodes and concluded with a novel graph-based collective classification algorithm. Recently Xu and Ashley (2022) deviated from classical sentence level classification in AM and has worked with the classification of argumentative text on token-level for legal text, where each token is a word. Xu and Ashley (2022) has created their dataset with BIO tagging scheme and annotated the legal case with the custom label; Issue, Conclusion, and Reason. Finally, they have approached the argument component classification task as a token classification task, which resulted in a better performance compared to sentence-level using Longformer (Beltagy et al., 2020). Habernal et al. (2023) introduced a new annotation scheme for ECHR comprising 16 categories for the argument and 4 for the actors (individuals/groups making the argument). They utilized the BIO scheme for annotation, reasoning that traditional argument models (for instance, claim/premise) are unsuitable for complex legal cases. Consequently, they have adopted Legal-BERT and RoBERTa-Large as the classification model and further pretrained RoBERTa-Large on English ECHR and JRC-Acquis-En (Steinberger et al., 2006) dataset. Notably, the latter model exceeded its predecessors.

In our study for AM in the legal domain, we have adopted two state-of-the-art LLMs, GPT-3.5 and GPT-4 (OpenAI, 2023). Unfortunately, there are limited technical details regarding these models (at the time of writing this paper) as they remain closed-source. Nevertheless, we have fragments of details about their current architecture, development, and how they progressed to their present state over time. The GPT (Radford et al., 2018) model was introduced as an autoregressive model grounded on the transformer network while only using the decoder block (Vaswani et al., 2017). The model followed was GPT-2 (Radford et al., 2019), an upgraded version of the previous model with 1.5B parameters compared to 117M. GPT-3 (Brown et al., 2020b) is an extension of GPT-2, has 175B parameters, and utilizes 45TB of text data for pre-training. GPT-3 has yielded excellent results, particularly in zero and few shot settings. Following in the footsteps of the success of GPT-3, the next iteration of this model was designed, InstructGPT (Ouyang et al., 2022). The significant innovation of this work is centered on utilizing alternative techniques for fine-tuning the GPT-3 model, particularly Reinforcement Learning from Human Feedback (RLHF) (Christiano et al., 2017), which allowed the model to follow human instruction better than earlier models. One of the most recent versions of InstructGPT is GPT-3.5 and its successor, GPT-4 (OpenAI, 2023), and our study mainly focuses on these two models. It is essential to note that GPT-3.5 and GPT-4 are pre-trained models we have adapted for inference only. Besides, the training data utilized to train these models is proprietary and held private by OpenAI, the developer of GPT-3.5 and GPT-4.

## 3 Materials and methods

### 3.1 Dataset description

The ECHR corpus was first presented in the light of argument mining by Moens et al. (2007) for identifying non-argumentative sentences, conclusions, and premises. Subsequently, it was extended in their subsequent works (Mochales and Moens, 2008, 2011) where the author has followed Walton's model for argumentation (Walton, 2009). Recently Poudyal et al. (2020) published an annotated corpus that contains 42 ECHR decisions and is likewise based on Walton's model (Walton, 2009), which includes 42 ECHR case-laws from Decision and Judgement categories. We characterize this corpus in this study as ECHR-AM to be consistent with prior work (Zhang et al., 2021; Lillis et al., 2022). ECHR dataset has also been used in other studies besides argument mining, for instance, to extract events from court decisions (Filtz et al., 2020) and judgment prediction (Chalkidis et al., 2019; Medvedeva et al., 2020).

**Figure 1:**
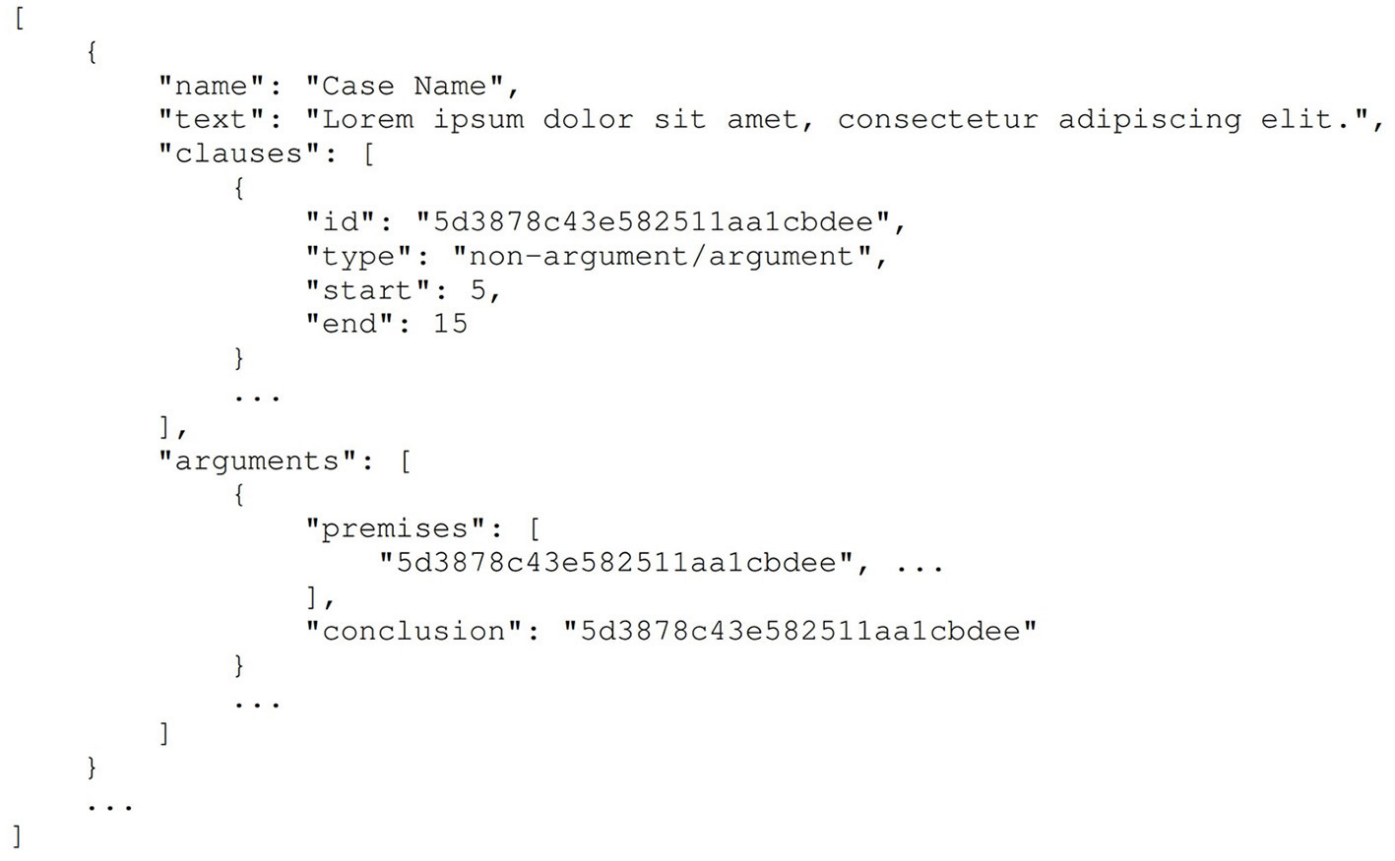
Data structure used for storing the ECHR-AM dataset by Poudyal et al. (2020). This Figure is reproduced under the Creative Commons Attribution 4.0 International License.

ECHR-AM consists of 20 decisions and 22 judgments case-laws, annotated following Walton's argumentation model (Walton, 2009). Four annotators were involved in this process, and the resulting Cohen's kappa inter-rater agreement of 0.80 was reached. Mochales and Moens (2008) provides a detailed structure of these case laws. Table 1 provides the statistics of the argument components. The dataset is delivered as a JSON file containing 42 cases following the structure, roughly, in Figure 1. Each case law has the field described in Figure 1, where the name field stores the case name and the text field contains the complete case law as text. Each case law has a list of clauses with a unique identifier, id, linked to it, along with the respective clauses' start and end character offset. The argument units (consisting of premises and a conclusion) are also presented similarly. Each argument component, arguments, (premise and conclusion) has a unique ID that maps to the clauses' id in the current case law, which refers to the text part of the case and eventually captures the content. The dataset does not precisely observe the schema presented in Figure 1; the type filed is not present in the dataset. Instead, the clause not part of any argument component is non-argumentative. It is critical to note that specific clauses can be a premise for a particular argument and a conclusion for another argument. Additionally, it is even possible for some argument components to function as premises/conclusions in multiple argument units. Lastly, the relation between each argument component is not annotated in the dataset; as an alternative, the authors have stored an argument unit as one item in the JSON file, which implicitly defines the support relation between the argument components.

**Table 1:** Statistics of argument component present at the document level for all cases.

	**Premise**	**Conclusion**
Minimum	8	4
Mean	47.74	18.29
Maximum	147	50
Total	1951	743

### 3.2 Argument mining task

In Poudyal et al. (2020); Zhang et al. (2021); Lillis et al. (2022), the authors have framed the argument-mining task as a pipeline that involves three distinctive stages for the ECHR-AM dataset. The first task is argument clause identification, which identifies whether a clause is argumentative or non-argumentative from the given corpus. The subsequent task in the pipeline is identifying the relation between the argument clauses. The final task in the pipeline is argument clause classification (which we focus on in this study), where a clause is identified as a conclusion or a premise. At its core, it is a sequence classification task. In this last stage of this pipeline, to simplify their (Poudyal et al., 2020; Zhang et al., 2021) experiments, they assumed that all the components had already been successfully identified in the previous steps. We frame this last task as an isolated task by itself and consider it a sequence classification problem. We believe this is reasonable given that the dataset is structured with explicit conclusion and premise annotation. Previous work approached this problem as a two separate binary classification task (Poudyal et al., 2020; Zhang et al., 2021; Lillis et al., 2022) since (as mentioned earlier in Section 3.1) a clause can be a premise for a given argument and can also be the conclusion for another argument. We hypothesize that these characteristics of the dataset will have a negative effect on the performance of GPT-3.5 and GPT-4. As we can anticipate, the prompt injected into these models might have more than one clause labeled as premise/non-premise in the premise identification task or vice versa for the conclusion identification task. This can trigger the model to unexpected behavior. However, to be consistent and comparable with our analysis across the literature, we also approach this task as a binary classification problem where we identify premise/non-premise and conclusion/non-conclusion. As a result of this design choice, we had to prompt the LLMs twice, which is expensive, given that GPT-3.5 and GPT-4 are behind a paywall.

### 3.3 Experimental design

In this section, we elaborate on our experimental setup. We have adopted two state-of-the-art GPT language models from OpenAI,^10^ gpt-3.5-turbo^11^ and the next generation model, gpt-4^12^ (OpenAI, 2023). We fixed the temperature for all our experiments to 0 (to confirm that the model behaves deterministically) while keeping the other parameters to default. It is important to note that the model can behave stochastically even after setting the temperature to zero.^13^ Therefore, it is impossible to guarantee these models' complete deterministic behavior. We added an extra instruction in the system message^14^ to ensure that the model generates with the argument component as we have prompted it. The system message for the premise classification task is *You must reply with either “premise” or “non-premise”*., and for the conclusion classification task is *You must reply with either “conclusion” or “non-conclusion”*.. GPT-3.5 series models tend not to follow the instructions sometimes (Blair-Stanek et al., 2023), which was also present in our early development stage.

We have followed the experimental setup proposed by Poudyal et al. (2020); Zhang et al. (2021); Lillis et al. (2022). We performed 5-fold cross-validation and split the dataset on the document level, not on the instance level. There are 42 case-laws in total in this dataset. In previous work, the authors have used 60% of the documents for training, 20% for validation, and 20% for testing. In each fold, they selected the model with the highest F1-score on the validation set and reported the result on the test set. We skip this part since we do not have a validation step in our experiment (at the time of writing, this is currently not possible with GPT-3.5 and GPT-4). However, for choosing samples as few-shots in the prompt, we select them from 60% of the train set for each fold to avoid data leakage, but unfortunately, we do not have information regarding the training data of GPT-3.5 or GPT-4; therefore, we can only control our data leakage. Other than that, we follow the experimental setup mentioned in prior work using the ECHR-AM dataset (Poudyal et al., 2020; Zhang et al., 2021; Lillis et al., 2022). For the evaluation metric, we followed the earlier work of Poudyal et al. (2020); Zhang et al. (2021); we have reported an average of standard precision, recall, and f1 score along with their standard deviation for 5-fold cross-validation. Since the dataset is heavily imbalanced, we omitted accuracy as well since it will be a misleading metric for performance measurement. Providing standard deviations for each metric will allow us to capture the stochastic nature of these LLMs and have an intuitive understanding of their consistency. Lillis et al. (2022) have deviated from the previous works of Poudyal et al. (2020); Zhang et al. (2021) that has performed the identical experiment and reported weighted evaluation metrics regarding precision, recall, and F1. They have argued this choice was made to capture the label imbalance in the dataset. To be consistent with the majority of the work in the literature and especially with the original work (Poudyal et al., 2020), we have decided to report on standard precision, recall, and F1 score. Following the experimental setup as before will allow us to compare the performance of our LLMs with the previous work.

**Figure 2:**
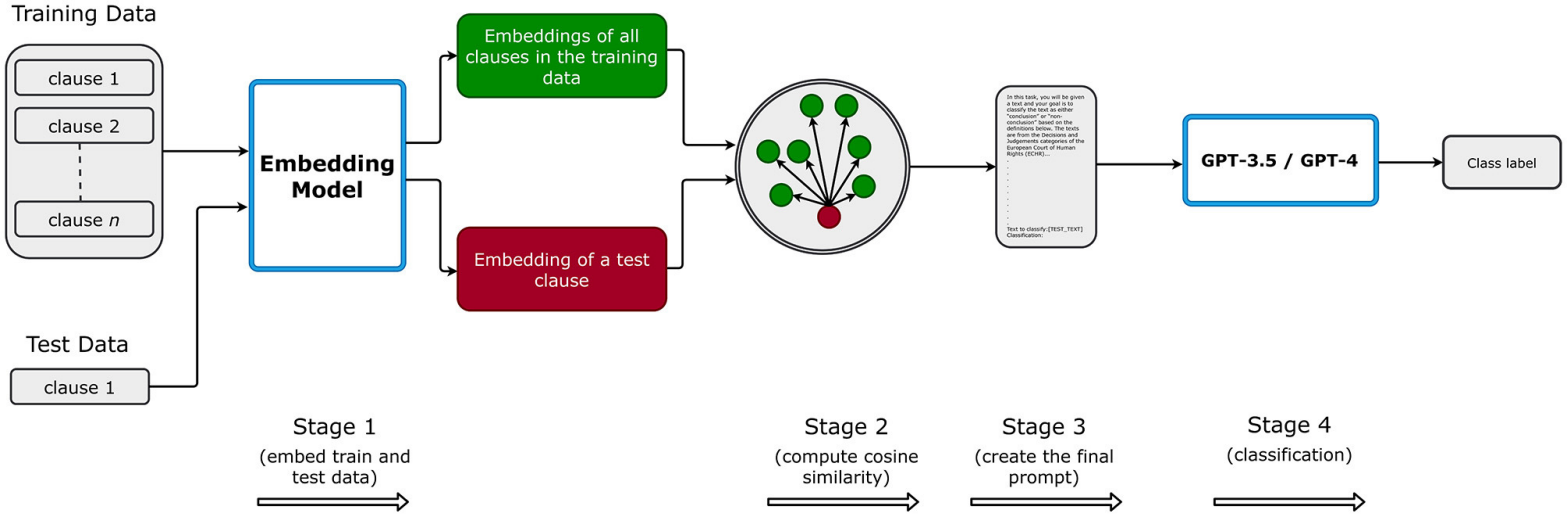
Illustration of our experimental setup. This Figure is reproduced under the Creative Commons Attribution 4.0 International License.

In Figure 2, we roughly illustrate our experimental process. First, in stage 1, we create embeddings for all training dataset instances in each fold and store them locally, and then, we embed the test text. Consequently, in stage 2, we compute the cosine similarity between the test example and all the train instances in the training set for the current fold. The cosine similarity ranges from -1 to 1, quantifying the degree of similarity between two embeddings. A cosine similarity value of 1 between two embeddings signifies complete similarity, and -1 signifies complete dissimilarity. In stage 3, based on the number of *n*-shot, we select *n* examples and their labels and append them to our prompt template. As mentioned in Section 3.4.3, for selecting similar and dissimilar examples in the prompt, we choose the top-*n* examples that have the highest and lowest cosine similarity between the test and all the train instances, respectively. For random selection, we select the examples randomly from all the train instances and skip Stage 1 and Stage 2. Likewise, we skip Stages 1 and 2 for zero-shot prompting and use the instruction to classify the argumentative clauses (from the test set); further information is in Section 3.4.3. Finally, in stage 4, we have a prompt with examples and labels (except for zero-shot prompting) and a test clause to be classified by the model. We repeat the process for each fold, i.e., five times, and report the mean metrics (precision, recall, and F1-score) along with their standard deviation.

We have benchmarked our result against the previous work of Poudyal et al. (2020) and Zhang et al. (2021). The authors in Poudyal et al. (2020) first introduced the annotated ECHR dataset and provided a baseline using RoBERTA (Liu et al., 2019). Subsequently, the authors in Zhang et al. (2021) have extended the previous work of Poudyal et al. (2020) using domain-specific encoder-based language models. They have adopted BERT (Devlin et al., 2018) based variants from Legal-BERT family (Chalkidis et al., 2020; Zheng et al., 2021) that are pre-trained on legal data. We refer the reader to Zhang et al. (2021) for further information.

### 3.4 Model details and design justification

#### 3.4.1 GPT language models

As mentioned in Section 2 we have adopted two LLMs, GPT-3.5 and GPT-4, for our study, which are accessible via OpenAI API.^15^ Provided that they are state-of-the-art autoregressive models that have already proven successful (Liu et al., 2023b; OpenAI, 2023) in various fields of NLP, we utilize these two models to evaluate their performance in argument mining, particularly in the field of law.

We also experimented with an open-source state-of-the-art GPT-like model, OpenAssistant LLaMA (Köpf et al., 2023), which is instruction-fine-tuned (Ouyang et al., 2022) on LLaMA-1 (Touvron et al., 2023a). In our early development phase, we observed that the model could not consistently follow instructions in the prompt. We hypothesize this alignment issue is inherently present in this model in contrast to GPT-3.5 or GPT-4, which are highly optimized toward following instructions. Therefore, we did not include it in our evaluation. However, one potential technique to mitigate this issue can be creating an instruction dataset (following our prompt format) based on the training dataset and instruct fine-tune the model. We speculate this would allow the model to better align with our prompt structure. We plan to investigate this line of research in the future.

#### 3.4.2 Embedding models

Word embeddings are a form of numerical representation in an n-dimensional vector that encodes the word's semantics that machines can utilize for further downstream tasks (Gasparetto et al., 2022). In our experiments, we have utilized two embedding models to select the number of n-shot examples with two (similar and dissimilar) settings. The two embedding models we include are

1. text-embedding-ada-002^16^ from OpenAI (which is behind a paywall, and the model is closed source) and
2. multi-qa-mpnet-base-dot-v1 model from the open-source framework of sentence transformers (Reimers and Gurevych, 2019).

The sentence-transformer framework provides several embedding models that are specialized and evaluated on different tasks. Based on the Model Overview^17^ in the sentence transformer documentation page, we have selected the model that has the best performance in semantic search, multi-qa-mpnet-base-dot-v1, since our use case aligns with this model. This model is based on the 6-layer version.^18^ of MiniLM (Wang et al., 2020) The model is trained with the objective of self-supervised contrastive learning.^19^ The embedding dimension is 768; the pooling method used CLS pooling, and the context size is 512. We have also used CLS pooling as default and adopted the model as it is from the sentence-transformer framework. multi-qa-mpnet-base-dot-v1 was trained using 215M question and answer pairs from different disciplines, including but not restricted to StackExchange, Yahoo Answers, WikiAnswers, and others; the details of the dataset utilized for training are available at this URL.^20^

Concerning text-embedding-ada-002, being a closed model, there are fewer details known. The official blog^21^ announcing the model reports that the embedding dimension is 1536, and the context length is 8,192. We have selected this embedding model to compare its performance with open-source embedding models.

Following our investigation of embedding and language models, we address the observed concerns affecting our final design choice. Due to budget constraints, our experiment with GPT-4 (which is 20 and 30 times more costly for input and output tokens, respectively, compared to GPT-3.5) is based on the configuration that led to the best overall performance of GPT-3.5. Nevertheless, GPT-3.5 provides a reliable baseline, indicating how the performance will deviate based on different settings. For selecting the best setting, we have considered the average F1-score of the conclusion and premise (from **Table 6** in Section 4), which indicate the model's overall performance. The highest average F1-score was achieved with the combination of the local embedding model, similar mode, and 8-shot example. Even though none of the highest scores belongs to the local-embedding model, the average performance is better. The average F1-score for the selected setting is 66.35%, whereas the next best score is 66.25% for the OpenAI embedding model with a similar 8-shot example.

#### 3.4.3 Prompt formulation

We follow the few-shot prompting strategy (Brown et al., 2020b) and couple it with instructions stating the purpose of the task (Ye et al., 2023). It is essential to note that GPT-3.5 and GPT-4 are fine-tuned to follow instructions, as they are the derivative model of InstructGPT (Ouyang et al., 2022) by OpenAI. Given this, it is appropriate to prompt the model using instruction, as this will align with the model's training technique. As discussed earlier, ICL is not robust against modifications (Zhao et al., 2021b) in the prompt. Liu et al. (2021a) confirmed that the example we select in the prompt as a few-shot influences the model's performance. Following this, we also investigate the robustness of our prompt. Even though the structure of law text is quite complex compared to any other text (for example, Liu et al. (2021a) evaluated on the sentiment analysis task), we anticipate finding a similar trend in our evaluation.

As mentioned earlier, prompts can be seen as hyperparameters that require tuning like any other hyperparameter in traditional machine-learning algorithms. However, due to the wide variety of prompts, the procedure and impact of prompt-hyperparameter tuning are less understood. For creating the instruction in our prompt, we followed Walton's argumentation model (Walton, 2009), which is also the basis of the annotation of the ECHR-AM dataset. Additionally, we consulted Mochales and Moens (2008), where the detailed structure of the ECHR corpus is given. The hyperparameters we have selected to tweak for the prompts are 1) the type of (similar, dissimilar, and random) examples to be added in few-shot prompting and 2) the number of examples *n*, (0,2,4, and 8); further details can be found in Section 3.3. We kept the instructions identical for each prompt to maintain consistency across our experiments. As mentioned in Section 3.3, we have prompted GPT-3.5 and GPT-4 twice for each argumentative component in mixed settings (following the previous work of Poudyal et al., 2020; Zhang et al., 2021; Lillis et al., 2022). An example of a complete prompt for the conclusion and premise recognition (excluding the few-shots examples) is provided in Tables 2, 3, respectively. We also provide the prompt we used for zero-shot in Tables 4, 5. To systematically evaluate our models, we standardized the prompt structure in all our experiments. The details of how few-shot examples are incorporated into the final prompt are in Section 3.3. Besides, please refer to Supplementary material for a detailed analysis of GPT-3.5's sensitivity to variations in instruction.

**Table 2:** Prompt for conclusion identification using few shot learning strategy.

**In this task, you will be given a text and your goal is to classify the text as either “conclusion” or “non-conclusion” based on the definitions below. The texts are from the Decisions and Judgements categories of the European Court of Human Rights (ECHR)**.
“conclusion”: In the context of argumentation in case law, a “conclusion” is the final decision or judgment made by the Commission or Court. It is often supported by one or more non-conclusions. The conclusion is the result of the argumentative process and is the central point that the argument is trying to establish.
“non-conclusion”: In the context of argumentation in case law, a “non-conclusion” refers to the statements, facts, or assertions that provide the basis/reason for a conclusion. They are the reasons given to support the final decision of the Commission or Court. They form the building blocks of the argumentative structure leading to the conclusion.
Below are examples of texts that are correctly classified as “conclusion”/“non-conclusion”.
[EXAMPLES WITH LABELS ARE INSERTED HERE]
Text to classify:[TEST_TEXT]
Classification:

**Table 3:** Prompt for premise identification using few shot learning strategy.

**In this task, you will be given a text and your goal is to classify the text as either “premise” or “non-premise” based on the definitions below. The texts are from the Decisions and Judgements categories of the European Court of Human Rights (ECHR)**.
“premise”: In the context of argumentation in case law, a “premise” refers to the statements, facts, or assertions that provide the basis/reason for a non-premise. They are the reasons given to support the final decision of the Commission or Court. They form the building blocks of the argumentative structure leading to the non-premise.
“non-premise”: In the context of argumentation in case law, a “non-premise” is the final decision or judgment made by the Commission or Court. It is often supported by one or more premises. The non-premise is the result of the argumentative process and is the central point that the argument is trying to establish.
Below are examples of texts that are correctly classified as “premise”/“non-premise”.
[EXAMPLES WITH LABELS ARE INSERTED HERE]
Text to classify:[TEST_TEXT]
Classification:

**Table 4:** Prompt for conclusion identification using zero-shot learning strategy.

**In this task, you will be given a text and your goal is to classify the text as either “conclusion” or “non-conclusion” based on the definitions below. The texts are from the Decisions and Judgements categories of the European Court of Human Rights (ECHR)**.
“conclusion”: In the context of argumentation in case law, a “conclusion” is the final decision or judgment made by the Commission or Court. It is often supported by one or more non-conclusions. The conclusion is the result of the argumentative process and is the central point that the argument is trying to establish.
“non-conclusion”: In the context of argumentation in case law, a “non-conclusion” refers to the statements, facts, or assertions that provide the basis/reason for a conclusion. They are the reasons given to support the final decision of the Commission or Court. They form the building blocks of the argumentative structure leading to the conclusion.
Text to classify:[TEST_TEXT]
Classification:

**Table 5:** Prompt for premise identification using zero shot learning strategy.

**In this task, you will be given a text and your goal is to classify the text as either “premise” or “non-premise” based on the definitions below. The texts are from the Decisions and Judgements categories of the European Court of Human Rights (ECHR)**.
“premise”: In the context of argumentation in case law, a “premise” refers to the statements, facts, or assertions that provide the basis/reason for a non-premise. They are the reasons given to support the final decision of the Commission or Court. They form the building blocks of the argumentative structure leading to the non-premise.
“non-premise”: In the context of argumentation in case law, a “non-premise” is the final decision or judgment made by the Commission or Court. It is often supported by one or more premises. The non-premise is the result of the argumentative process and is the central point that the argument is trying to establish.
Text to classify:[TEST_TEXT]
Classification:

## 4 Results

### 4.1 Analysis of prompt structure

In this Section, we present the result of our analysis of using GPT-3.5 for argument component classification, considering various prompt configurations and utilizing semantic search.

Table 6 presents the evaluation of GPT-3.5 and GPT-4 under diverse settings. In this Section, we focus only on GPT-3.5, as mentioned before in Section 3.3; due to budget constraints, our experiment with GPT-4 is based on the best configuration that led to the highest performance by using GPT-3.5. Embedding model represents which model was used in the semantic search for few-shot prompting in the range of 2,4 and 8, where two, four, and eight texts, along with their labels, are provided as few-shot examples. The mode defines the type of semantic search we have adopted for example selection in few-shot prompting; the modes we have chosen to investigate are similar, dissimilar, and random. Zero-shot prompting, i.e., when *N* is zero, does not contain any examples in the prompt; hence, the embedding model and the mode of prompt selection do not apply. Furthermore, zero-shot prompting acts as a baseline for the rest. Similarly, the embedding model does not apply to the random mode, as the example selection is done randomly. We report each component's precision, recall, and f1 score in each individual setting, along with the standard deviation. At the bottom of Table 6, we also present the baseline results from Zhang et al. (2021) for comparison. Since we investigate the performance of GPT models (GPT-3.5 and GPT-4) by tweaking various settings, below we have structured them for convenience and compactness.

**Table 6:** Experimental result for the argument component classification.

**Language model**	**Embedding model**	**Mode**	**N-shots**	**Precision(%)**		**Recall(%)**		**F1-score(%)**	
				**Premise**	**Conclusion**	**Premise**	**Conclusion**	**Premise**	**Conclusion**
GPT-3.5	Not Applicable	Not Applicable	0	74.3 ± 3.2	**79.2 ± 2.6**	61.6 ± 3.6	18.2 ± 6.7	67.3 ± 2.8	29.0 ± 8.5
	multi-qa-mpnet-base-dot-v1	Similar	2	76.0 ± 4.0	69.7 ± 2.7	94.8 ± 2.0	36.5 ± 6.8	84.3 ± 3.0	47.5 ± 6.0
			4	76.4 ± 3.7	68.9 ± 3.2	95.4 ± 2.1	34.6 ± 6.0	84.8 ± 2.5	45.7 ± 4.9
			8	76.1 ± 3.8	70.9 ± 3.4	96.4 ± 2.0	36.6 ± 8.3	85.0 ± 2.6	47.7 ± 6.5
		Dissimilar	2	75.5 ± 3.5	69.2 ± 2.2	86.8 ± 1.0	35.8 ± 8.8	80.7 ± 2.2	46.5 ± 7.5
			4	76.5 ± 3.5	68.3 ± 4.4	81.7 ± 1.3	36.3 ± 7.5	79.0 ± 2.2	47.0 ± 6.6
			8	76.8 ± 3.6	67.6 ± 3.0	80.2 ± 3.1	37.2 ± 7.0	78.4 ± 3.0	47.7 ± 6.1
	Not Applicable	Random	2	75.9 ± 3.5	69.9 ± 1.5	90.7 ± 2.5	36.9 ± 8.1	82.6 ± 2.7	47.8 ± 6.9
			4	76.4 ± 3.4	71.9 ± 2.6	88.4 ± 2.9	35.5 ± 6.8	82.0 ± 2.7	47.0 ± 5.7
			8	76.7 ± 3.4	71.6 ± 1.6	90.9 ± 2.1	34.2 ± 7.1	83.1 ± 2.5	45.8 ± 6.3
	text-embedding-ada-002	Similar	2	75.9 ± 3.7	68.9 ± 4.2	95.7 ± 1.2	35.8 ± 7.4	84.7 ± 2.5	46.7 ± 6.6
			4	76.2 ± 4.1	68.3 ± 3.3	96.3 ± 1.4	34.3 ± 6.1	85.0 ± 2.6	45.3 ± 4.9
			8	75.9 ± 4.3	69.4 ± 3.0	**96.8 ± 1.2**	36.5 ± 6.6	85.1 ± 2.8	47.4 ± 5.2
		Dissimilar	2	76.3 ± 3.5	70.1 ± 1.9	89.1 ± 2.8	39.7 ± 7.9	82.1 ± 2.7	50.1 ± 6.1
			4	77.2 ± 3.4	68.1 ± 3.5	84.9 ± 3.0	40.0 ± 8.4	80.8 ± 2.5	49.9 ± 6.8
			8	76.9 ± 3.3	68.2 ± 3.1	85.6 ± 3.1	40.9 ± 7.2	81.0 ± 2.7	50.9 ± 6.1
GPT-4	multi-qa-mpnet-base-dot-v1	Similar	8	**80.0 ± 3.8**	61.5 ± 3.5	91.5 ± 3.4	**45.7 ± 7.7**	**85.3 ± 2.5**	**51.9 ± 4.2**
Baseline (Zhang et al., 2021)	Not Applicable	Not Applicable	Not Applicable	83.9 ± 1.6	67.1 ± 0.9	94.6 ± 2.3	67.2	87.2 ± 0.6	64.2 ± 1.8
(best score)				(Legal-BERT_harv_)	(C-Legal-BERT_harv_)	(BiLSTM)	(RoBERTa)	(C-Legal-BERT_harv_)	(Legal-BERT_harv_)

Reporting mean of precision, recall, and F1-score along with standard deviation for 5-fold cross-validation. The bold values indicates the highest score achieved by GPT-3.5/4 model in argument component classification tasks for precision, recall and f1-score.

One widespread trend we can observe for the *premise* classification task is as the number of examples increases, the F1-score also increases. There is a clear correlation between the number of examples in the prompt with the model's performance. However, this trend is not observed when the examples are dissimilar; the F1-score drops from 80.7% to 78.4% and from 82.1% to 81.0% when using local and OpenAI embedding models, respectively. However, when the examples are chosen randomly, there is no clear trend: the F1-score decreases from 82.6% to 82.0% and then increases again to 83.1% for two, four, and eight examples, respectively. The highest F1-score (for premise recognition), 85.1%, was gained using OpenAI embedding with eight similar examples in the prompt. Nevertheless, the local embedding model was also on par with the OpenAI embedding model and had an F1-score of 85.0%. From this observation, we can conclude that including examples in the prompt that are semantically similar has a favorable impact on the performance of the GPT-3.5 model for the premise recognition task. One explanation for this behavior is that having seen examples similar to the test instances allows the model to align itself for predicting the correct label more often.

The highest precision for premise can be observed using dissimilar mode with eight examples using OpenAI embeddings, 76.9%, and the lowest precision, 74.3%, in zero-shot settings. The highest recall was observed for a similar eight-shot example using OpenAI embedding, 96.8%, and the lowest in the zero-shot setting, 61.6%. Even though this observation for precision and recall is contradictory for the premise class, as the highest and lowest score, respectively, was achieved by similar and dissimilar examples, it is clear that the number of examples provided in the prompt plays an essential role in improving the model's performance.

Regarding the *conclusion* classification task, we observe a dramatic drop in the F1-score (lowest 29.0%) compared to the premise (lowest 67.3%). This was expected since the dataset is highly imbalanced with 1951 premises and 743 conclusions. Regardless of the low F1-score, we can observe an opposite behavior regarding the kind of example leading to the highest F1-score for the conclusion class. The highest F1-score, 50.9%, was gained using eight dissimilar examples. This is the opposite regarding the type of example (similar) that led to the highest F1-score for the premise class. The recall of the conclusion class is relatively very low, lowest 18.2%, compared to the recall, lowest 61.6%, of the premise class. Nevertheless, the recall increases to 40.9%, by using eight dissimilar examples. Interestingly, the precision of the conclusion class is highest, 79.2%, in a zero-shot setting but compromising recall (lowest 18.2%).

In summary, we can observe that prompt tuning plays an essential role in guiding the model's performance. On average, the performance of GPT-3.5 (regarding F1-score) is better when eight examples are chosen semantically, and the model delivers a worse performance in zero-shot setting. The performance drop in the zero-shot setting can be attributed to the model relying entirely on its training knowledge to infer the class label. Whereas in a few-shot setting, the model can learn via ICL from the examples. From this, we can reason that the most similar examples in the prompt will guide the model to serve better. Finally, we observe a slight improvement in the performance of GPT-3.5 when the local model is used for creating the embedding, compared to OpenAI embedding model (66.35% F1 score on average for both the class using the local embedding model, compared to 66.25% using OpenAI embedding model). We can infer that the embeddings from the local model are as semantically rich (and even slightly better) as the OpenAI embeddings.

### 4.2 Analysis of model's performance

The baseline models demonstrate the most substantial performance across all the metrics (except for precision for the conclusion and recall for the premise), surpassing GPT-3.5 and GPT-4. The F1-score for the premise and conclusion using GPT-4 is 85.3% and 51.9% compared to 87.2% by C-Legal-BERT_harv_ and 64.2% by Legal-BERT_harv_, respectively. We do not observe a significant increase in the performance metrics of GPT-4 compared to GPT-3.5 considering the F1-score, with a gain of 0.2% and 1%, for premise and conclusion classes, respectively. Only in the precision of the conclusion class (79.2%) and the recall of the premise class (96.8%), GPT-3.5 exceeds the baseline model's performance.

We hypothesize this happened for two main reasons: 1) As mentioned in Section 3.3, we have modeled the argument component classification task into two binary classification tasks [to be consistent with the literature (Poudyal et al., 2020; Zhang et al., 2021; Lillis et al., 2022)]. Since some clauses can be a premise for an argument and a conclusion of another argument, or it is possible that some clauses function as premises/conclusions in multiple arguments. Therefore, we can observe that when adapting the dataset to be suitable for the binary classification tasks, there might be a clause labeled as both conclusion and non-conclusion or premise and non-premise. It is important to note that this characteristic of the data will still prevail even if we discard the aforementioned modeling approach. This indicates the necessity of altering the modeling technique to suit the dataset better.

As mentioned earlier, ICL is not robust to minor changes in the prompt (Zhao et al., 2021b) regarding structure, type of training samples, or their order. We hypothesize that this characteristic of the data heavily biases the model toward predicting the wrong label. For instance, when the model is presented with a text with two contradictory labels, it is very unlikely that it will be able to learn from the context it is exposed to. 2) Legal texts are inherently complex in semantics and structure. In Poudyal et al. (2020), the author had to consult with third lawyers after a low kappa inter-rater agreement (0.58) between the first two lawyers to analyze the reason for this low score and with the fourth lawyer performed the annotation again (which increased the inter-rater agreement to 80%). From this, it is evident that legal data are complex, even for lawyers. We hypothesize that this complexity has a negative impact on the model's performance. Lastly, we might also benefit from sanitizing the prompt and the test text, allowing the model to adjust to the context better. We performed our experiment without text preprocessing to be consistent with the literature.

From our aforementioned observation regarding GPT-3.5's and GPT-4's performance being lower than the baseline models, we further analyze the prompt and data in Section 4.2.1 and Section 4.2.2, respectively.

#### 4.2.1 Prompt analysis

To keep our analysis concise, we delve deeper into the experiment with GPT-4 only. We are more interested in identifying the reason behind GPT-4's low performance compared to GPT-3.5 since GPT-4 has demonstrated to be highly successful in diverse NLP tasks, without the need for special prompting, surpassing ChatGPT (Bubeck et al., 2023).

We begin our analysis by randomly selecting a complete prompt given to GPT-4 in the first fold. As mentioned in Section 3.3, we have carried out five-fold cross-validation on all our experiments; therefore, we also hold information concerning the input and output of all instances in each fold. First, we pick a complete prompt used to classify the conclusion class. In Table 7, we provide the complete prompt. We observed that two texts are repeated in the prompt with opposite labels, conclusion and non-conclusion. The first example text, *This part of the application must accordingly be rejected under Article 26 in conjunction with Article 27 para. 3 (Art. 26+27-3) of the Convention as having been introduced out of time*., has the label conclusion and non-conclusion. To confirm our finding, we delve into the raw ECHR dataset, which is in JSON format. We have successfully identified that, indeed, this aforementioned text has been annotated with two labels, conclusion, and non-conclusion (as mentioned in Section 3.3, we approached the task as two binary classification tasks, and hence, a premise is considered as non-conclusion for premise classification task; this was done to maintain consistency in the literature).

**Table 7:** A complete input to GPT-4 for conclusion classification task including few-shot examples and the test text.

**In this task, you will be given a text and your goal is to classify the text as either “conclusion” or “non-conclusion” based on the definitions below. The texts are from the Decisions and Judgements categories of the European Court of Human Rights (ECHR)**.
“conclusion”: In the context of argumentation in case law, a “conclusion” is the final decision or judgment made by the Commission or Court. It is often supported by one or more non-conclusions. The conclusion is the result of the argumentative process and is the central point that the argument is trying to establish.
“non-conclusion”: In the context of argumentation in case law, a “non-conclusion” refers to the statements, facts, or assertions that provide the basis/reason for a conclusion. They are the reasons given to support the final decision of the Commission or Court. They form the building blocks of the argumentative structure leading to the conclusion.
Below are examples of texts that are correctly classified as “conclusion”/“non-conclusion”.
Example:According to the Government, the applicants failed to exhaust their domestic remedies
Classification:conclusion
Example: According to the Government, the applicant
herself was responsible for the delays in
Classification:conclusion
Example:This part of the application must accordingly be rejected
under Article 26 in conjunction with Article 27 para. 3 (Art. 26+27-3)
of the Convention as having been introduced out of time.
Classification:non-conclusion
Example:This part of the application must accordingly be rejected
under Article 26 in conjunction with Article 27 para. 3 (Art. 26+27-3)
of the Convention as having been introduced out of time.
Classification:conclusion
Example: It follows that the applicant did not have access to a “tribunal”.
Classification:conclusion
Example: It follows that the applicant did not have access to a “tribunal”.
Classification:conclusion
Example: It follows that the applicant did not have access to a “tribunal”.
Classification:non-conclusion
Example: It follows that the applicant did not have access to a “tribunal”.
Classification:non-conclusion
Text to classify: The respondent Government considers that the applicant submitted his application out of time
Classification:

Subsequently, the second example text, *It follows that the applicant did not have access to a “tribunal”*. is present four times in the prompt as examples, and two times labeled as conclusion and other two times it is labeled as non-conclusion. Similarly, as before, we examined the raw ECHR dataset to verify this finding. We have again found that this text was indeed annotated with a conclusion and premise in multiple cases (and interestingly in six places in total, three times premise and three times conclusion).

Secondly, we select a complete prompt employed to classify the premise class (in the same fold, i.e., first). In Table 8, we provide the complete prompt. We observe a similar pattern as before with the conclusion class. Two texts are repeated in the prompt with opposite labels, premise and non-premise. The first example text, *The Government submitted that Mr Ahmet Sadik had not exhausted domestic remedies, not having raised before the national courts, even in substance, the complaint relating to a violation of Article 10 (art. 10)*., has the label premise and non-premise. The second example text, *Accordingly, the Court considers that the reasons given by the national authorities for the measures taken in respect of the applicants were relevant and sufficient for the purposes of Article 8 para. 2 (art. 8-2)*. is present three times as an example, and two times as non-premise and once as a premise. We again examined the raw ECHR dataset and verified our findings in a similar way as before. We also performed the same analysis for the conclusion and premise class for prompts in fold five and observed a similar pattern. We include these results in Supplementary material.

**Table 8:** A complete input to GPT-4 for premise classification task including few-shot examples and the test text.

**In this task, you will be given a text and your goal is to classify the text as either “premise” or “non-premise” based on the definitions below. The texts are from the Decisions and Judgements categories of the European Court of Human Rights (ECHR)**.
“premise": In the context of argumentation in case law, a “premise" refers to the statements, facts, or assertions that provide the basis/reason for a non-premise. They are the reasons given to support the final decision of the Commission or Court. They form the building blocks of the argumentative structure leading to the non-premise.
“non-premise”: In the context of argumentation in case law, a “non-premise" is the final decision or judgment made by the Commission or Court. It is often supported by one or more premises. The non-premise is the result of the argumentative process and is the central point that the argument is trying to establish.
Below are examples of texts that are correctly classified as “premise"/“non-premise".
Example: The Government submitted that Mr Ahmet Sadik had not exhausted domestic remedies, not having raised before the national courts, even in substance, the complaint relating to a violation of Article 10 (art. 10).
Classification:premise
Example: The Government submitted that Mr Ahmet Sadik had not exhausted domestic remedies, not having raised before the national courts, even in substance, the complaint relating to a violation of Article 10 (art. 10).
Classification:non-premise
Example: The Court notes that the applicant's insecure personal circumstances arising from the loss of his home does not fall within the notion of security of person as envisaged by Article 5 § 1 of the Convention (see the Selçuk and Asker v. Turkey, Commission's report, cited above, § 186).
59. In the light of the foregoing, the Court concludes that there has been no violation of Article 5 § 1 of the Convention.
Classification:non-premise
Example:There is no doubt that these acts, in addition to giving rise to a violation of Article 3, constituted a grave and unjustified interference with the applicant's rights to respect for his private and family life and home, and to the peaceful enjoyment of his possessions (see Menteş and Others v. Turkey, judgment of 28 November 1997, Reports 1997-VIII, p. 2711, § 73, and Dulaş v. Turkey, no. 25801/94, § 60, 30 January 2001).
Classification:premise
Example: Accordingly, the Court considers that the reasons given by the national authorities for the measures taken in respect of the applicants were relevant and sufficient for the purposes of Article 8 para. 2 (art. 8-2).
Classification:non-premise
Example: Accordingly, the Court considers that the reasons given by the national authorities for the measures taken in respect of the applicants were relevant and sufficient for the purposes of Article 8 para. 2 (art. 8-2).
Classification:non-premise
Example:Accordingly, the Court considers that the reasons given by the national authorities for the measures taken in respect of the applicants were relevant and sufficient for the purposes of Article 8 para. 2 (art. 8-2).
Classification:premise
Example:the Court considers that, in the circumstances of the present case, there are grounds for examining the applicant's other complaints under that Article (see mutatis mutandis, Göç v. Turkey [GC], no. 36590/97, § 46, ECHR 2002-V).
Classification:non-premise
Text to classify:In respect of damage alleged to have been caused by the State or
its agents, the Government submit that the applicants had the
possibility of introducing an action for compensation before the civil
or administrative courts relying, inter alia, on Article 125 of the
Turkish Constitution or Article 8 of Decree 430 of 16 December 1990.
Classification:

We can expect that this data characteristic has a decisive role in lowering GPT-4's performance; likewise, we can infer that the effect is similar for GPT-3.5. As mentioned earlier, when the model is presented with several texts with opposite ground truths, it is reasonable to conclude that this will degrade its performance. We would also like to point out that. This is an intrinsic feature of the ECHR-AM dataset (Poudyal et al., 2020); an argumentative clause can be both a conclusion and a premise (Poudyal et al., 2020). This is also a general characteristic of an argument, where a conclusion can act as a premise of another argument. and a premise can act as a conclusion for another argument.

#### 4.2.2 Data analysis

**Figure 3:**
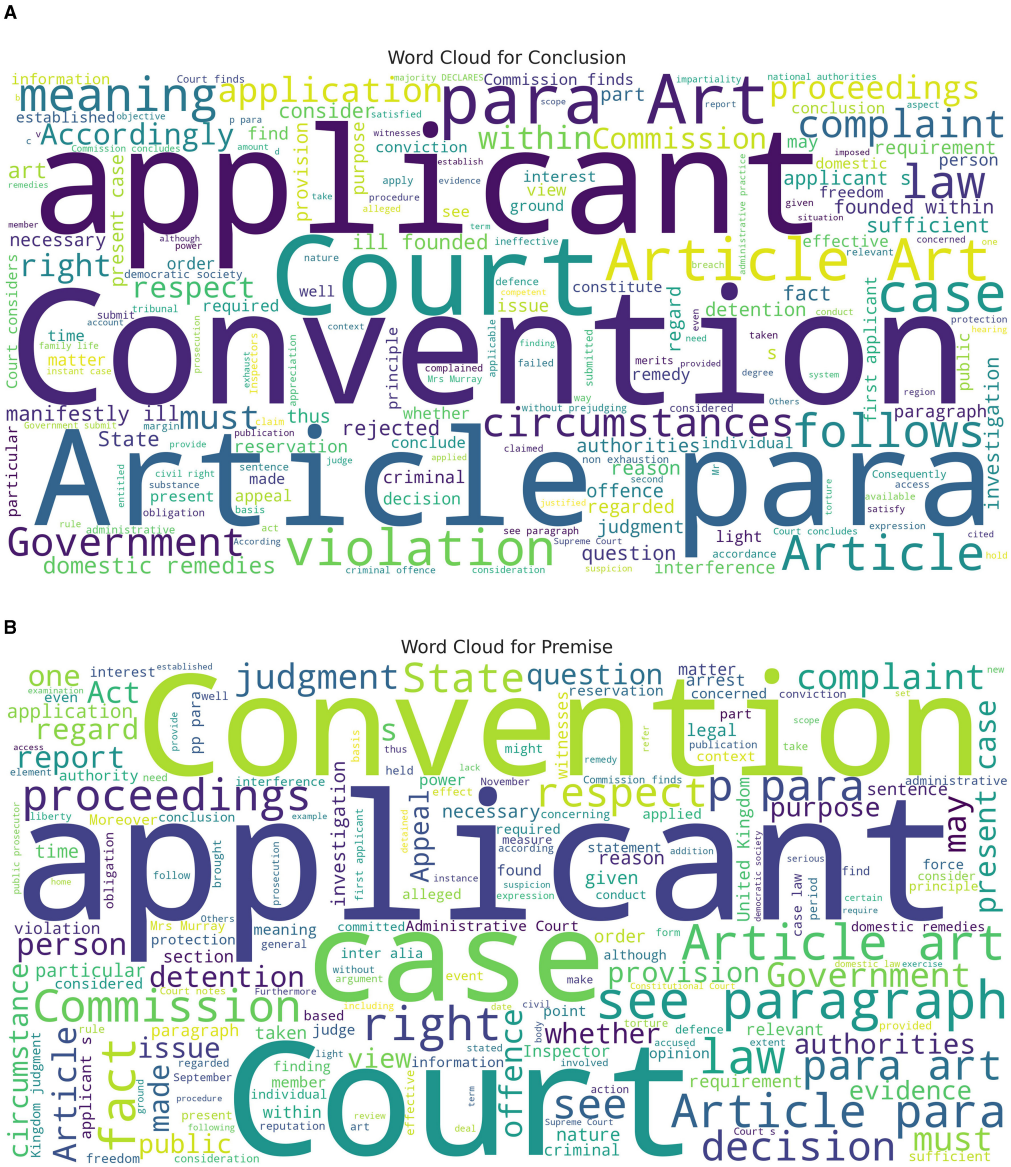
Word cloud for the conclusion and premise class. **(A)** Conclusion class word cloud. **(B)** Premise class word cloud. This Figure is reproduced under the Creative Commons Attribution 4.0 International License.

In this section, we analyze the data further and speculate to have more insight into GPT-3.5's and GPT-4's lower performance than the baseline. Figure 3 displays the word cloud for both the conclusion and premise classes. We can observe that the most frequent word in the text for conclusion and premise classes overlaps heavily. For example, the top most frequent words in both classes are: applicant, Convention, and Court. Given the high overlap of the most frequent word, we hypothesize that it is challenging for GPT-3.5 and GPT-4 to differentiate between these two classes.

**Figure 4:**
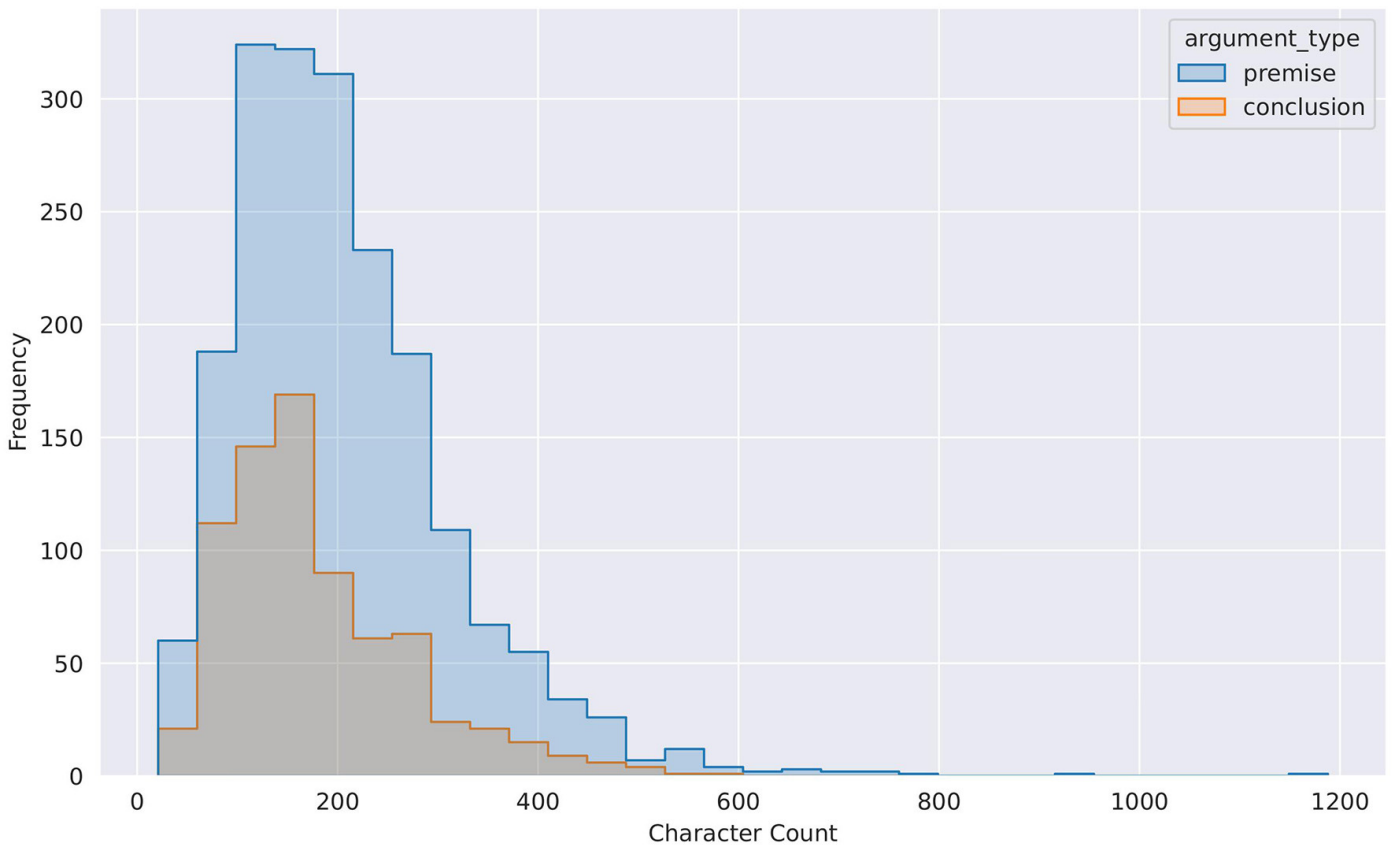
Character count distribution for conclusion and premise classes. This Figure is reproduced under the Creative Commons Attribution 4.0 International License.

Furthermore, Table 9 provides summary statistics based on the argument components' length (character-based). One intriguing observation that we can make from Table 9 is that the span of an argument component has a significant variance. Besides, we also observe that the conclusion and premise class' length can go as low as 21 and 26 characters, respectively. Figure 4 graphically displays the distribution of the text's length (conclusion and premise) based on characters.

**Table 9:** Statistics of the length of the argument on character level.

**Character based**	**Premise**	**Conclusion**
Maximum length	1188	583
Minimum length	26	21
Mean length	205.08	179.34
Standard Deviation	109.42	91.39

Generative models like GPT-3.5 and GPT-4 rely on their context, and through ICL (Dong et al., 2023), they can learn and perform various NLP tasks. We hypothesize that the span of the argument component also played an essential role (but less prominent than examples with conflicting labels) in these models' performance and had a negative impact on our experimental result. The models might not have enough context to differentiate between premises and conclusions due to the shorter length of these argument clauses.

## 5 Discussion

ChatGPT and GPT-4 have demonstrated significant performance in diverse disciplines, including, but not limited to, the legal domain (Yu et al., 2022; Blair-Stanek et al., 2023; Katz et al., 2023a; Savelka et al., 2023). As mentioned earlier, argumentation plays a vital role for legal professionals, such as lawyers (Palau and Moens, 2009). Lawyers present their opinions structured and argumentatively to defend their statements. To better understand their view, it is essential to determine the rationale that ultimately favors their standpoint. Argument mining is the automated approach for determining and extracting the argument elements and their structure from texts written in natural language. Despite the recent success of these language models, it is yet to be realized in the domain of law for argument mining. In this study, we analyzed the performance of GPT-3.5 and GPT-4 for classifying argument components in the ECHR-AM dataset. We have also systematically varied the prompt to quantify the model's sensitivity regarding prompt formulation.

We have empirically found that, on average, the performance of the baseline models (Poudyal et al., 2020; Zhang et al., 2021) surpasses GPT-3.5 and GPT-4. We hypothesize this materialized given how we have modeled the classification task into binary classification (following previous work in the literature), leading to examples labeled with both the classes in the samples, which hampers the model's performance. We have conducted an in-depth analysis of the prompt to verify our hypothesis. Indeed, there are argument clauses labeled as both conclusion and non-conclusion or premise and non-premise. This is an inherent characteristic of the ECHR-AM dataset; an argumentative clause can be both a conclusion and a premise (Poudyal et al., 2020). Furthermore, we investigated if the text length of the argument units can be an additional factor for the low performance of GPT-3.5 and GPT-4. After delving into the dataset, we discovered that each argument unit's length has high variance, and the conclusion and premise class's length can go as low as 21 and 26 characters, respectively. We hypothesize that this also biased these models performance and had an unfavorable influence on our experimental outcome. Nevertheless, unlike the baseline models, GPT-3.5 and GPT-4 are not finetuned and rely solely on prompts and examples given to the model. Finetuning PLMs or LLMs is computationally expensive, given that during the process, the model's parameters are updated. On the other hand, we have utilized GPT-3.5 and GPT-4 via prompting only, without updating the model's parameter.

Given the low support for the conclusion class in the ECHR dataset, we observe a substantial difference in F1-score compared to the premise recognition task. This, unfortunately, is an inherent characteristic of any argumentation (Palau and Moens, 2009), where many premises support a conclusion. We also observe that prompt tuning is critical in guiding the GPT models for the best performance. Our finding of the prompt sensitivity aligns with prior research (Liu et al., 2021a; Zhao et al., 2021b). Selecting the appropriate example via semantic search and the number of examples for few-shot learning can significantly increase or decrease the model's performance. We have also empirically learned that the embeddings from the local model are on par with the embedding from OpenAI models, which guides toward the direction of adopting the local model for semantic search is more favorable than accessing the OpenAI model that is behind a paywall.

In this study, we have analyzed the performance of GPT-3.5 and GPT-4, considering diverse prompt formulations using semantic search for argument component classification tasks. However, this study has a couple of limitations. We did not include several latest prompting strategies in our study, for example, chain-of-thought prompting (Wei et al., 2023) its variants (Shum et al., 2023; Weng, 2023). The maximum number of examples in the prompt we have adopted is eight. Increasing the number of examples might influence the performance of these models positively. Since every prompt feeds some bias to these models, we hypothesize that our prompt also biased the model in a certain way. Changing the prompt, especially removing the instructions, will provide a better understanding of these models' reliance on prompt design. Recently Llama-2 (Touvron et al., 2023b), an open-source GPT-like model, has been released by Meta, which we have not included in our study. We have adopted two pre-trained embedding models not tailored to the legal domain. Observing the superior performance of domain-specific models on legal data, it will be interesting to fine-tune local embedding models using legal data and benchmark their performance against domain-agnostic models.

## Data availability statement

Publicly available datasets were analyzed in this study. This data can be found here: http://www.di.uevora.pt/~pq/echr.

## Author contributions

AA: Writing – original draft, Writing – review & editing. MG: Writing – review & editing. JM: Writing – review & editing.

## Footnotes

^1^ https://github.com/features/copilot

^2^ https://platform.openai.com/docs/models/gpt-3-5

^3^Official blog:https://openai.com/blog/chatgpt ChatGPT was fine-tuned from a GPT-3.5 series model, but it is not explicitly cited which model ChatGPT is founded on.

^4^ https://openai.com/blog/chatgpt

^5^ http://www.di.uevora.pt/~pq/echr/

^6^ https://huggingface.co/sentence-transformers/multi-qa-mpnet-base-dot-v1

^7^ https://openai.com/blog/new-and-improved-embedding-model

^8^ https://zenodo.org/record/8246129

^9^ https://hudoc.echr.coe.int/eng

^10^ https://platform.openai.com/docs/models/overview

^11^gpt-3.5-turbo belongs to the series GPT-3.5 model.

^12^We refer to gpt-3.5-turbo as GPT-3.5 and gpt-4 as GPT-4, for brevity and syntactic consistency.

^13^ https://platform.openai.com/docs/models/gpt-3-5

^14^ https://platform.openai.com/docs/guides/gpt/chat-completions-api

^15^ https://platform.openai.com/docs/api-reference

^16^ https://openai.com/blog/new-and-improved-embedding-model

^17^ https://www.sbert.net/docs/pretrained_models.html#model-overview

^18^ https://huggingface.co/nreimers/MiniLM-L6-H384-uncased

^19^ https://huggingface.co/sentence-transformers/multi-qa-MiniLM-L6-dot-v1#background

^20^ https://huggingface.co/sentence-transformers/multi-qa-MiniLM-L6-dot-v1#training

^21^ https://openai.com/blog/new-and-improved-embedding-model
