# Supplementary material for: Performance analysis of large language models in the domain of legal argument mining
Source: Front Artif Intell. 2023 Nov 17;6:1278796. doi: 10.3389/frai.2023.1278796 (PMC10691378; doi:10.3389/frai.2023.1278796)
Supplement: Supplementary file 1 [file Data_Sheet_1.PDF]

Supplementary Material

1 EXTENDED PROMPT ANALYSIS

In this section, we have extended our analysis of the complete input to GPT-4. We have randomly selected a complete prompt for the conclusion classification task given to GPT-4 in the fifth fold, present in Table S1. We observe that the text, "*Nonetheless, the manner in which findings detrimental to a person's reputation are arrived at in an administrative investigation, as well as the objectives pursued by the investigation, is relevant for assessing the permissibility under Article 6 para. 1 (art. 6-1) of a limitation on the person's opportunities to go to court to enforce his or her civil right to reputation.*" is repeated twice in the prompt with the opposite label, once with non-conclusion and other times with a conclusion. We again delved into the original ECHR dataset and confirmed our observation. We obtain a similar pattern for premise classification prompts in Table S2. The text, "*The Court therefore finds that the applicant was subjected to indirect and improper pressure to make statements in respect of his application to the Commission, which interfered with the free exercise of his right of individual petition guaranteed under Article 25.*" is repeated twice in the prompt with the label of premise and non-premise.

Table S1. A complete input to GPT-4 for premise classification task including few-shot examples and the test text.

In this task, you will be given a text and your goal is to classify the text as either ”conclusion” or ”non-conclusion” based on the definitions below. The texts are from the Decisions and Judgements categories of the European Court of Human Rights (ECHR).

”conclusion”: In the context of argumentation in case law, a ”conclusion” is the final decision or judgment made by the Commission or Court. It is often supported by one or more non-conclusions. The conclusion is the result of the argumentative process and is the central point that the argument is trying to establish.

”non-conclusion”: In the context of argumentation in case law, a ”non-conclusion” refers to the statements, facts, or assertions that provide the basis/reason for a conclusion. They are the reasons given to support the final decision of the Commission or Court. They form the building blocks of the argumentative structure leading to the conclusion.

Below are examples of texts that are correctly classified as ”conclusion”/”non-conclusion”.

Example:In the present case the
applicant did not object to the constitution of the court-martial and
in any event, according to the Government, it does not follow from the
fact that the members of the court-martial were all subordinate in rank
to the Convening Officer (having no direct prior relationship with that
Officer) that they lacked independence or impartiality.

Classification:non-conclusion

Example:It is necessary not only to recognise the discretion of the competent domestic court in maintaining conformity with the exigencies of the proper administration of justice, but also to balance the interests of the defence under Article 6 (art. 6) against the interests of witnesses protected by other substantive provisions of the Convention (see the Doorson judgment previously cited, p. 470, para. 70).
Classification:non-conclusion

Example:The Court therefore concludes that there has been a violation of Article 8 of the Convention and of Article 1 of Protocol No. 1.
Classification:conclusion

Example:It is
submitted that it is a generally recognised principle of international
law that an accused person cannot be required to incriminate himself,
that Article 6 (Art. 6) of the Convention enshrines this principle, and
that the drawing of an incriminating inference from an accused's
failure to give evidence infringes his right to a fair trial.

Classification:non-conclusion

Example:Rather their claim was that where a report by organs of the State has branded an individual as being guilty of wrongdoing such as dishonesty after a procedure not attended by the procedural guarantees of a fair trial, Article 6 para. 1 (art. 6-1) grants the individual whose reputation is at stake the right to challenge the findings against him or her in a court of law before publication of the report.
Classification:non-conclusion

Example:The Court therefore concludes that there has been a violation of Article 3 of the Convention.
Classification:conclusion

Example:Nonetheless, the manner in which findings detrimental to a person's reputation are arrived at in an administrative investigation, as well as the objectives pursued by the investigation, is relevant for assessing the permissibility under Article 6 para. 1 (art. 6-1) of a limitation on the person's opportunities to go to court to enforce his or her civil right to reputation.
Classification:non-conclusion

Example:Nonetheless, the manner in which findings detrimental to a person's reputation are arrived at in an administrative investigation, as well as the objectives pursued by the investigation, is relevant for assessing the permissibility under Article 6 para. 1 (art. 6-1) of a limitation on the person's opportunities to go to court to enforce his or her civil right to reputation.
Classification:conclusion

Text to classify:The Court considers that in situations where a court is faced with misbehaviour on the part of any person in the courtroom which may constitute the criminal offence of contempt, the correct course dictated by the requirement of impartiality under Article 6 § 1 of the Convention is to refer the question to the competent prosecuting authorities for investigation and, if warranted, prosecution, and to have the matter determined by a different bench from the one before which the problem arose.
Classification:

Table S2. A complete input to GPT-4 for premise classification task including few-shot examples and the test text

In this task, you will be given a text and your goal is to classify the text as either ”premise” or ”non-premise” based on the definitions below. The texts are from the Decisions and Judgements categories of the European Court of Human Rights (ECHR).<br><br>”premise”: In the context of argumentation in case law, a ”premise” refers to the statements, facts, or assertions that provide the basis/reason for a non-premise. They are the reasons given to support the final decision of the Commission or Court. They form the building blocks of the argumentative structure leading to the non-premise.<br><br>”non-premise”: In the context of argumentation in case law, a ”non-premise” is the final decision or judgment made by the Commission or Court. It is often supported by one or more premises. The non-premise is the result of the argumentative process and is the central point that the argument is trying to establish.<br><br>Below are examples of texts that are correctly classified as ”premise”/”non-premise”.<br><br>Example: The applicants contended that the Inspectors’ investigation and, above all, the publication of the Inspectors’ report gave rise to a violation of Article 6 para. 1 (art. 6-1), which, in so far as relevant, provides:<br>Classification:premise<br><br>Example:It follows that the applicant did not have access to a ”tribunal”. There has accordingly been a violation of Article 6 para. 1 (art. 6-1) on this point.<br>Classification:non-premise<br><br>Example: Concerning the first applicant, the Court recalls its finding under Article 5 § 3 (see paragraph 103) that he was released before any judicial control of his detention would have been feasible<br>Classification:premise<br><br>Example:The Commission notes that the applicant’s conviction involved his writings.<br>Classification:premise<br><br>Example:It is not possible to establish in detail what happened during the applicant’s meeting with the public prosecutor, but the Commission finds no reason to doubt that during their conversation there were elements which should have made the public prosecutor initiate an investigation or, at the very least, try to obtain further information from the applicant about his state of health or about the treatment to which he had been subjected.<br>Classification:premise<br><br>Example:Turning to the facts of the instant case, the Court observes that it is accepted by the parties that the applicant was indeed interviewed by the public prosecutor on 6 June 1995 as alleged upon the instructions of the Ministry of Justice International Law and Foreign Relations Directorate concerning his application with the Commission (see paragraphs 39 and 56).<br>Classification:premise<br><br>Example: The Court therefore finds that the applicant was subjected to indirect and improper pressure to make statements in respect of his application to the Commission which interfered with the free exercise of his right of individual petition guaranteed under Article 25.<br>Classification:premise<br><br>Example: The Court therefore finds that the applicant was subjected to indirect and improper pressure to make statements in respect of his application to the Commission which interfered with the free exercise of his right of individual petition guaranteed under Article 25.<br>Classification:non-premise<br><br>Text to classify:The applicant refers here in particular to the Public Prosecutor’s order<br>of 12 August 1992 which, as a result of the applicant’s article<br>published on 11 August 1992, prohibited him from transmitting written<br>materials to his lawyers.<br>Classification:
