# Supplementary material for: Performance analysis of large language models in the domain of legal argument mining
Source: Front Artif Intell. 2023 Nov 17;6:1278796. doi: 10.3389/frai.2023.1278796 (PMC10691378; doi:10.3389/frai.2023.1278796)
Supplement: Supplementary file 2 [file Data_Sheet_2.pdf]

Supplementary Material

1 GPT-3.5'S SENSITIVITY TO PROMPT STRUCTURE

In this section, we study the potential impact of prompt structure on the performance of the GPT-3.5 model. Notably, we alter the prompt by removing "The texts are from the Decisions and Judgements categories of the European Court of Human Rights (ECHR)" only. The final prompt is identical to Table 2 and Table 3, excluding the phrase. This permits us to quantify the influence of including or excluding the phrase on GPT-3.5's performance and identify potential bias in our prompt. Due to budget constraints, we experimented with GPT-3.5 only. For the experimental setup, we have adopted the setting that led to the best performance of GPT-3.5, i.e., using the local embedding model ("multi-qa-mpnet-base-dot-v1") and semantically similar eight examples in the prompt. Nonetheless, we consider this to provide a reasonable understanding of the GPT model's sensitivity towards prompt structure.

Table S1 presents the evaluation of GPT-3.5 on two prompt settings. The notable difference between Table S1 and Table 6 is the inclusion of the "Phrase" column, where "Excluded" indicates that the phrase "The texts are from the Decisions and Judgements categories of the European Court of Human Rights (ECHR)" was not included in the prompt and "Included" signifies the phrase was included in the prompt. A general observation that we can make from our result is that both prompt types yield an almost similar result. We make the following observations based on the prompt that does not include the particular phrase. We notice no significant difference in the F1-score for the premise and conclusion tasks, with an increase of 0.4% and 1.5%, respectively. Similarly, the recall is also alike in both prompt types, with an increase of 0.3% for the premise and 2.7% for the conclusion classes. However, we observe an opposite trend for precision, with a decrease of 0.8% and 3.9% for the premise and conclusion classes, respectively.

Nevertheless, we notice no discernible distinction in the performance of GPT-3.5 between the two prompt settings based on F1-score, recall, and precision. This slight variation in the model's performance suggests that including the phrase "The texts are from the Decisions and Judgements categories of the European Court of Human Rights (ECHR)" does not bias the model significantly. Although current budget limitations restrict us from further investigating GPT-4 (and similarly with all other configurations with GPT-3.5), we see it as a promising future work.

Table S1. Experimental result for argument component classification using GPT-3.5 with and without ECHR dataset mentioned in the prompt. Reporting precision, recall, and F1-score along with standard deviation for 5-fold cross-validation

Language Model	Embedding Model	Mode	N-shots	Phrase[a]	Precision(%)		Recall(%)		F1-score(%)	
					Premise	Conclusion	Premise	Conclusion	Premise	Conclusion
GPT-3.5	multi-qa-mpnet-base-dot-v1	Similar	8	Excluded	75.3±4.2	67.0±4.0	96.7±1.9	39.3±5.7	84.6±2.7	49.2±4.1
				Included[b]	76.1±3.8	70.9±3.4	96.4±2.0	36.6±8.3	85.0±2.6	47.7±6.5

[a] "The texts are from the Decisions and Judgements categories of the European Court of Human Rights (ECHR)"
[b] This result is from our previous experiment presented in Table 6.
